# Interbrain synchronization in classroom during high-entropy music listening and meditation: a hyperscanning EEG study

**DOI:** 10.3389/fnins.2025.1557904

**Published:** 2025-04-01

**Authors:** Junling Gao, Hang Kin Leung, Kin Cheung (George) Lee, Chun Chi Poon, Gan Huang, Junhao Liao, Bonnie Wai Yan Wu, Thuan Quoc Thach, Rainbow Tin Hung Ho, Hin Hung Sik

**Affiliations:** ^1^Centre of Buddhist Studies, The University of Hong Kong, Hong Kong SAR, China; ^2^Buddhist Wong Wan Tin College, Hong Kong SAR, China; ^3^School of Biomedical Engineering, Shenzhen University, Shenzhen, China; ^4^Centre on Behavioral Health, The University of Hong Kong, Hong Kong SAR, China; ^5^Department of Psychiatry, The University of Hong Kong, Hong Kong SAR, China

**Keywords:** EEG, meditation, high-entropy music, brain synchronization, adolescence, hyperscanning, social connectedness

## Abstract

**Introduction:**

Social interaction is a vital source of human development, yet neuroscientific research delineating its neural correlates in large groups is scarce. Music as a rhythmic signal, and meditation, have been shown to induce group synchronization and pro-social behavior. However, their impact on adolescents may vary, and the related brain functions remain underexplored. This study investigates the effects of mindfulness meditation and 6 Hz high-entropy music on brain synchronization and complexity in high school students.

**Methods:**

Twenty-eight adolescents underwent single-channel EEG at the forehead during three 5-minute conditions: rest, meditation, and 6 Hz high-entropy music. Alpha band power correlations assessed synchronization. Graph analyses quantified network properties.

**Results:**

Mean correlation was highest during music, then meditation, and lowest during rest, with significant differences between music and both rest and meditation. Meditation had the highest clustering coefficient and small-world index, suggesting more integrated and efficient networks. Music demonstrated the largest information cascades and synergy, indicating extensive information integration.

**Conclusion:**

6 Hz high-entropy music induced the strongest synchronization. While meditation and music altered brain dynamics compared to rest, they worked distinctly. Meditation yielded more integrated connectivity; music yielded the greatest element-wise correlation. Future research with larger samples is recommended to optimize interventions for adolescent well-being and social connectedness.

## 1 Introduction

Pro-sociality is essential skills that need adolescence to explore and learn effectively (Bannan and Harvey, 2025). A majority of human knowledge is learned through interaction and discussion in society, where knowledge and information can flow effectively and coherently among individuals. Trust and connection are prerequisites for social connectedness, and there is always a need to explore and foster these conditions from the early stage of human life. Adolescence is a transformative process, marked by cognitive, emotional, and social changes (Steinberg, 2014). The brain undergoes significant reorganization, facilitating advanced abilities, emotional regulation, and complex social behaviors (Crone and Dahl, 2012). Due to these drastic and multifaceted changes, adolescents face many physiological, social, and personal challenges that can elevate stress, anxiety, and depression (Yu and Du, 2022). Unsuccessful coping with such challenges can lead to severe consequences, such as severe mental illness, self-injurious behaviors, and suicide (Wong and Chan, 2019). Previous studies suggested that interventions that cultivate resilience, enhance emotional regulation, and promote social connectedness are crucial in helping adolescents cope with different emotional distress (Rosenberg et al., 2021). However, such interventions are uncommon in Hong Kong which is a city with high rate of depression and suicide in adolescents (Leung and Mu, 2022).

Music as a rhythmic information has an inherent ability to coordinate our physical and psychological coherence, and beyond. Some music can easily synchronize the physical and feelings of a large number of people. Given this nature, music can be a cost effective and efficient way to enhance well-being and social connectedness in adolescents. On the other hand, relaxation and let-go attachment and self-guard is another prerequisite for social connection, and mind–body practice skills such as meditation can train individuals to be more relaxed and less stressed. Meditation involves focusing on the present, cultivating awareness, and developing non-judgmentality (Hölzel et al., 2013). It has been associated with reduced stress, improved attention, and enhanced emotional regulation (Zeidan et al., 2010; Kuyken et al., 2013). And in the education setting, meditation reduces stress and improves concentration (Waters et al., 2015). Regardless of the success of mindfulness meditation and music in promoting social coherence, little is known about the mechanism underlying, from a neuroscientific perspective.

Music’s impact on pro-social behavior is attributed to its effect on emotions. In another recent study, Wu and Chien (2024) showed music-induced positive emotions lead to increased altruism. Neural entrainment through music may contribute to positive states, promoting pro-social behavior. Understanding how music and meditation affect the human brain can help us to find the most effective way of music listening or meditation to enhance pro-sociality. After all, social connectedness is basically the brain interaction among individuals – the Hebrian learning rule for neuron connection in the brain network is ‘fire together, wire together’. The possibility exists that when people in a group are stimulated by the same stimuli, and this externally driven synchrony may eventually foster social connection.

Among the common brain oscillation band, theta frequencies (~6 Hz) rhythm can entrain neural oscillations and induce specific states (Colzato et al., 2017). For example, the frontal midline theta rhythm is often associated with the hippocampal theta rhythms. The frontal midline theta density is negatively correlated with scores in the anxiety scale, and found to be correlated with lower anxiety levels after anti-anxiety treatment (Kropotov, 2009). We thus assume that the theta rhythm of high-entropy music may help entrain neural oscillations, inducing states conducive to relaxation and improved pro-sociality when listening together. Specific rhythmic beat may entrain neural oscillations and induce specific states (Reedijk et al., 2015; Colzato et al., 2017). Also, we found that the auditory high-entropy response (high-entropy music) induced by 6 Hz beats increases neural responsiveness and processing at an individual level (Huang et al., 2021). We assumed that 6 Hz high-entropy music may help with inter-brain synchronization, which potentially could induce group coherence in this hyperscanning EEG study.

Binaural beats can increase theta and alpha oscillations, and the alignment of oscillations at theta band may facilitate relaxation and performance (Gao et al., 2014). Abundant EEG studies have shown that meditation increases alpha and theta, indicating relaxation (Lomas et al., 2015). Neural synchronization at these theta and alpha bands facilitates cognitive and emotional functions (Deco et al., 2015), and enhanced synchronization can further improve memory, and emotional regulation (Fell and Axmacher, 2011).

However, the existing research primarily focus on the individual level. Similarly, in the context of pro-social behavior, most neuroscientific studies focus on individual effects while neglecting their dynamic connectivity, likely constrained by the difficulties of hyperscanning setup in real world settings like classrooms. In particular, adolescents are underrepresented and there is a lack of research in real-world education, where the network complexity and information dynamics are much more complicated than brain processing at individual level. The interplay of synchronization and complexity at group level is also underexplored. Together with the alarming mental health problems, it is critical to develop more efficient and effective interventions for the next generation.

To address existing gaps, this study employs hyperscanning EEG to investigate the effects of high-entropy music and meditation on inter-personal synchronization and network complexity in students. Hyperscanning facilitates the examination of inter-brain connectivity and collective dynamics (Babiloni and Astolfi, 2014), offering valuable insights into social interactions within educational settings (Grootjans et al., 2024). While meditation and music may have a cooperative effect in fostering pro-sociality, this preliminary study would delineate their differential neural mechanisms. By comparing meditation and high-entropy music, this study also aims to delineate their distinct neural correlates and implications for adolescent well-being and social connection. It investigates how these interventions influence synchronization, complexity, and high-entropy music compared to rest. Recent studies have highlighted the importance of neural synchrony in social interactions, emphasizing its role in understanding the neural basis of social coordination (Grasso-Cladera et al., 2024). Our findings can elucidate neural mechanisms underlying meditation and auditory stimulation, providing neuroscientific evidence and potentially theoretic models for interventions aimed at improving mental health and social connectedness in adolescents.

## 2 Methods

### 2.1 Participants

Twenty-eight healthy, grade 12 high school students (13 males, 10 females, 5 with missing data; age 17 ± 1 years old) were recruited from a local school. All participants had no history of neurological or psychiatric disorders. Informed consent was obtained from both the participants and their parents, adhering to the ethical standards of the institutional review board. Additionally, the participants were amateur meditators who would practice meditation during their Buddhist study class. Participants completed a General Health Questionnaire (GHQ) that aimed to measure their general mental health and it has good sensitivity to their levels of stress, depression, and anxiety.

### 2.2 Procedure

As a group, each participant underwent EEG recording during three distinct conditions: 5 minutes of rest, 7 minutes of mindfulness meditation (with analysis based on the first 5 minutes), and 5 minutes of listening to 6 Hz high-entropy music. The aim of a group setting is to evaluate whether a collective practice of meditation and music listening plays a role in neurological effect. The entire setup and experiment were completed within a 40-min timeframe during the Buddhist study class.

- Rest condition: Participants were instructed to sit quietly with their eyes closed, maintaining a relaxed posture without engaging in any specific mental activity.
- Meditation condition: Participants engaged in a guided mindfulness meditation session, focusing on their breath and observing thoughts without judgment.
- Music condition: Participants listened to 6 Hz modulated music of Dream d’Amour delivered through speakers, the beats were designed to induce theta wave activity associated with relaxation and meditative states.

To ensure efficiency, all EEG headsets and phones were paired next to each seat beforehand. Students sat cross-legged in their assigned seats. A brief introduction about the experiment procedure was given, after which students cleaned their forehead areas with alcohol pads before wearing the devices. Researchers then checked the signal and impedance before starting the recordings. Throughout the entire experiment, the classroom teacher guided the students to ensure smooth execution.

### 2.3 EEG recording

EEG data were collected using a single-channel EEG device (UmindLight, EEGsmart) placed at the left forehead (Fp1 position) to monitor frontal brain activity. Previous study found that the left prefrontal cortex synchronized the most between the students and teacher in the classroom, thus assumerable suitable for hyperscanning study in classroom (Liu et al., 2019). The sampling rate was set at 256 Hz, and data were recorded with a reference electrode behind the right ear (Tp10 position). Impedances were kept low using gel to ensure signal quality as displayed on the control center smartphone and each individually paired smartphone. The SoulMirror app was used to create a virtual room so all the students’ EEG signal can be used to join the virtual room, check the signal quality and start data collection and end it altogether. Data were collected on the respective paired smartphones first, and then send out to the server, where it could be downloaded for further data analysis. Data were first inspected manually. EEG data with too much noise (20 *SD*) were replaced by artifact subspace reconstruction (ASR) method from EEGLAB toolbox. Data were filtered between 1 Hz and 40 Hz to remove artifacts and noise.

### 2.4 Data analysis

Data analysis was conducted using MATLAB, incorporating the EEGLAB toolbox for graph theoretical analyses. Twenty-six participants’ EEG datasets were analyzed, two datasets were excluded due to poor quality. The following steps were undertaken:

#### 2.4.1 Preprocessing

EEG data were segmented into 1-s epochs for each condition to mitigate the time lag effect of the internet and Bluetooth connections. These technical issues also led to minor missing data for some participants (< 5% in total). We inspected each dataset manually and used the previous seconds to fill in the missing data. A majority of the EEG datasets were accurately timed, as verified by their data length. The data for the meditation condition were shortened to match the other conditions.

Data were then filtered between 1 Hz and 40 Hz to remove artifacts and noise. Subsequently, the data were plotted as a time series for further inspection. Power spectral was obtained using EEGLAB’s spectopo function for each 1-s epoch. Dataset with the typical signatures of EEG, such as higher alpha and lower gamma power, were used as primary indicators of usable data (see Supplementary Figures 1–3).

EEG data with excessive noise (±20 SD) were corrected using EEGLAB’s Artifact Subspace Reconstruction (ASR) function before further analysis. The alpha power of each 1,000 ms data segment was calculated for further data analysis.

#### 2.4.2 Correlation matrices

Pearson’s correlation coefficients were computed between all pairs of participants using single-channel EEG signals to create correlation matrices for each condition (rest, meditation, music). Given the single-channel setup, synchronization metrics were derived based on temporal correlations. This element-wise method provided a fundamental estimate of the overall correlation. The mean and standard deviation of the upper triangular elements of each correlation matrix (325 *r*-values) were computed to summarize synchronization levels across conditions.

The questionnaire data GHQ (M = 17.35, SD = 5.16) was collected prior to the EEG data collection, no significant correlation between the GHQ scores and EEG data of alpha power was found.

#### 2.4.3 Statistical analysis

Paired t-tests were performed to examine differences in group synchronization between conditions: (1) Music vs. Rest, (2) Music vs. Meditation, and (3) Meditation vs. Rest. To stabilise the variances of the *r* values, we transformed the 325 *r*-values to *z*-values using Fisher’s z transformation for each condition. Next, we computed the differences between conditions—specifically, scores of Music minus Rest, Meditation minus Rest, and Music minus Meditation. Finally, these differences in scores were used to assess whether they deviated significantly from zero. To correct for these 3 multiple comparisons, we applied the False Discovery Rate (FDR) correction to the test using the Benjamini-Hochberg procedure.

#### 2.4.4 Graph theoretical analysis and small-world index calculation

Graph metrics, including the clustering coefficient, node degree, and small-world index (SWI) were calculated to assess the network properties of brain synchronization patterns in each condition. The SWI was calculated by comparing the clustering coefficient and average shortest path length of the observed networks against those of randomized networks. For the clustering coefficient calculated by the clustering_coef_bu function of the Brain Connectivity Toolbox, a binary value is generated for each of the 325 *r*-values in each condition; a value of 1 is assigned if the absolute value of r exceeds the threshold of 0.1. To ensure reproducibility, the same randomized parameters were used across all conditions (set to 1 for simplicity). Correlation matrices and network graphs were visualized to illustrate the patterns of synchronization and the presence of significant differences between conditions.

## 3 Results

### 3.1 Synchronization differences

**Figure 1:**
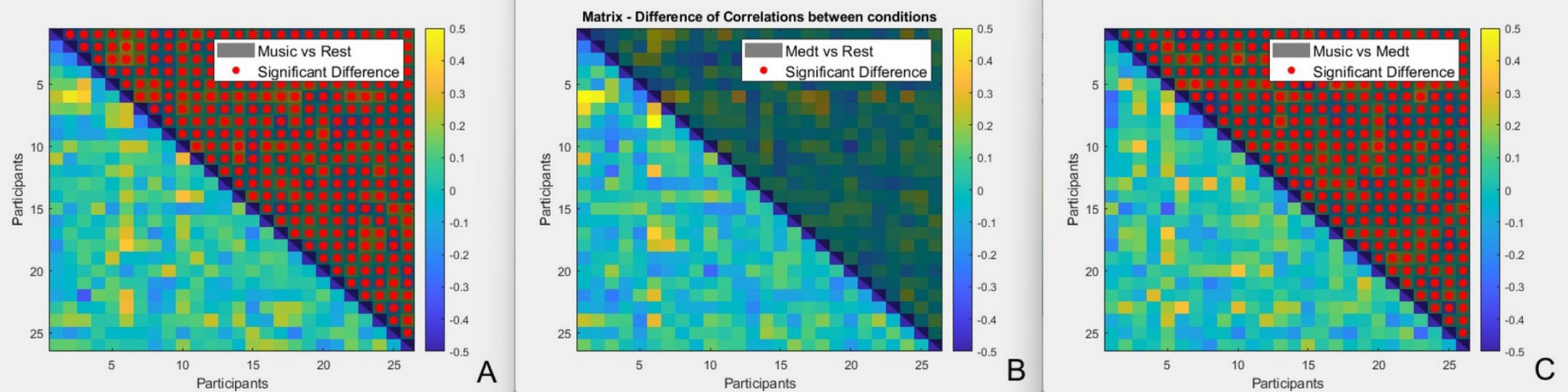
Group analysis on correlation matrix (element-wise). The color indicates the r difference between conditions **(A)** music and rest; **(B)** meditation and rest; and **(C)** music and meditation. Shaded areas and red dots represent the t-tests and their significance (*p* < 0.05, FDR corrected).

Element-wised comparison was made between correlation values of each pair under conditions of high-entropy-music listening, breathing meditation and normal rest. *p*-values were FDR corrected (see Figure 1). Paired t-tests revealed significant differences in brain synchronization between the music condition and both rest (*p* < 0.001) and meditation (*p* < 0.001), but no significant difference between meditation and rest (*p* > 0.05). For comparison between the three conditions, Fisher’s z-transformation was applied on the *r*-values first.

Specifically, the mean correlation was highest during the music condition (M = 0.0495 ± 0.0910), followed by meditation (M = 0.0197 ± 0.0952), and lowest during rest (M = 0.0114 ± 0.0802).

### 3.2 Graph theoretical metrics and small-world index

**Figure 2:**
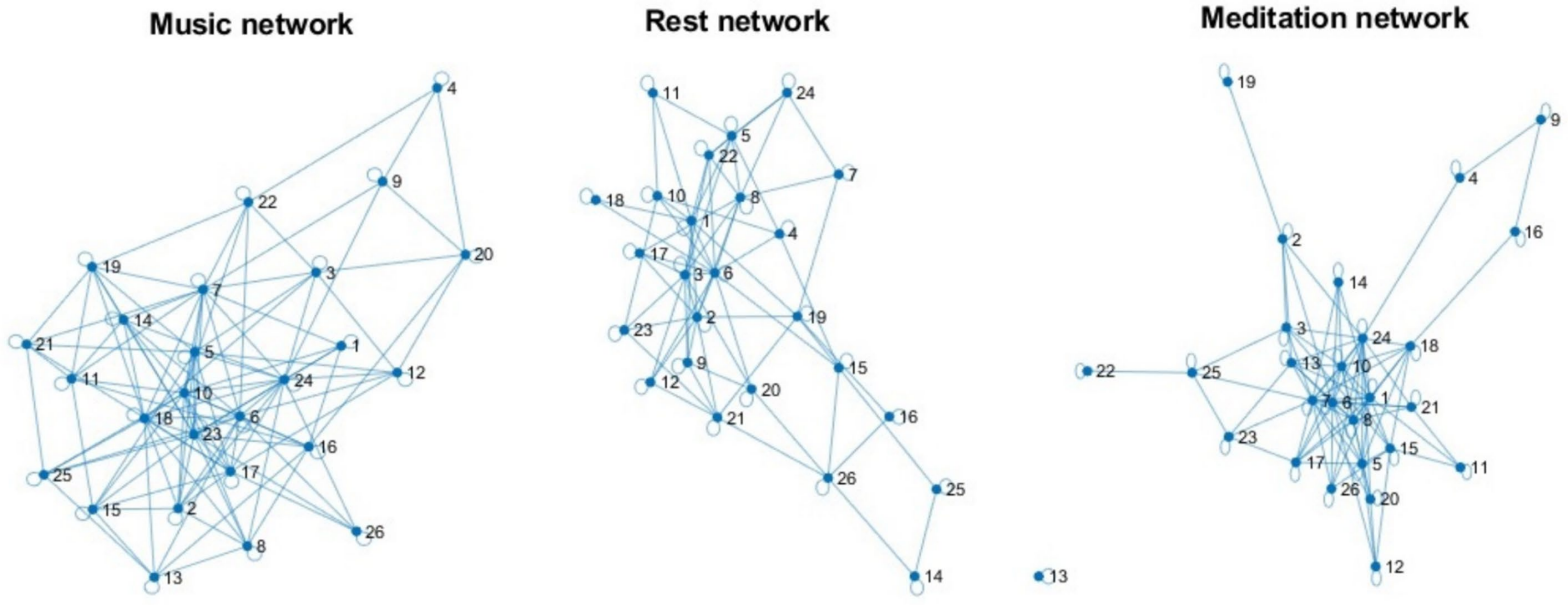
Depicts three brain network graphs corresponding to different experimental conditions—high-entropy music listening (left), normal rest (center), and breathing meditation (right). Each node in the network represents a student’s prefrontal alpha power, and each edge represents the strength of synchronization (i.e., functional connectivity) between two students’ brain activity.

Graph analysis indicated that the meditation condition exhibited the highest clustering coefficient (0.9437), suggesting more locally integrated and globally efficient neural networks. The small-world index was highest for meditation (SWI = 0.4691), followed by music (SWI = 0.4465), and rest (SWI = 0.3912). This indicates that meditation fosters a more small-world-like network topology, characterized by high clustering and short path lengths (see Figure 2).

## 4 Discussion

Music has long been regarded as a social instrument in history (Bannan and Harvey, 2025), while the underlying neural mechanism is rarely studied, due to the difficulty in simultaneously monitoring brain activities in a group of people. This study pioneered in exploring inter-brain connectivity in a relatively large group of students in real-world situation, and we demonstrated that high-entropy music could induce group synchronization in terms of alpha band power fluctuation and high node degree in graph theory analysis. Meditation, as another traditional way to induce transcendent feelings among a group, did not show a significant effect on group synchronization, but exhibited a trend of high SWI and clustering coefficient.

Our previous research on 6 Hz (theta band) auditory stimuli on brain response (Huang et al., 2021). Theta (4–8 Hz) plays a role in memory, emotion regulation, and creativity (Faber et al., 2021). This current study further found high-entropy music of 6 Hz had the strongest effect on inter-brain synchronization at alpha band. While 6 Hz music at theta band was chosen as stimulate waves, we chose to focus on alpha band power (8–12 Hz) for the specific analysis of inter-brain connectivity and group coherence. Alpha waves are widely regarded as a most obvious and prominent brain activity, and they are known to be critical for measuring synchronization between individuals, especially in group dynamics. It is highly sensitive to social interactions and collective brain states, making it a well-established frequency for studying inter-brain connectivity during group tasks (Dikker et al., 2017).

Higher group-based rhythmic activities promote interpersonal neural coherence, which may subsequently induce collective identity and emotional resilience. A solid finding is the significant increase in mean network synchronization during high-entropy music compared to rest and meditation. This aligns with literature suggesting this high-entropy music entrain oscillations associated with relaxation and meditative states (Jirakittayakorn and Wongsawat, 2017). Enhanced synchronization during music may facilitate coherent neural communication, underpinning cognitive and emotional benefits.

Our wearable EEG devices measure the brain activity at the left prefrontal theta, crucial for emotion regulation, cognitive control, and social processing (Huang et al., 2021). Previous study has found that the left prefrontal cortex is most responsive the coherent brain activity during teaching and learning in the classroom (Liu et al., 2019). This technique of modulating classical music to the one with high-entropy may enhance synchronization and complexity during music listening, leading to more robust social and emotional benefits.

We also found that high-entropy music condition induced the highest degree (number of connections) in nodes among the participants in the group, followed by meditation and rest conditions. According to the Hebbian theory on learning, fire together, wire together’, we assume that by promoting synchronized activity, high-entropy music can facilitate shared states, enhancing empathy, understanding, and coordinated behavior. This enhanced connectivity through synchronized stimulation may also improve communication and collective problem-solving, given the dynamic interaction between students. Thus said, it is worth noting that Hebbian learning is for neurons’ connection instead of persons’ connection. Confirming this trend requires careful measurement of behavioral scores alongside brain activity. Nevertheless, exploring this direction is relevant in educational and therapeutic settings, where effective communication and coordinated efforts are essential. Our findings contribute to research on music and pro-social behavior and is in line with previous study that the synchronous nature of group music listening is linked to pro-social behavior (McWeeny et al., 2024). McWeeny et al. found synchronous and anti-phase drumming elicited similar pro-social ratings, suggesting shared experience, rather than precise synchronization, may be the key in fostering pro-social tendencies. Enhanced group synchronization during music aligns with this, highlighting music’s potential to promote bonding and pro-social behavior.

Meditation did not significantly differ in overall synchronization compared to rest. However, graph analyses indicated meditation fostered a denser small-world network topology, with an indication of highest clustering coefficient and small-world index. High clustering indicates greater local interconnectedness, facilitating efficient processing within specialized clusters. The elevated small-world index reflects optimal balance of local specialization and global integration, essential for complex functions (Sporns, 2021). These properties suggest, while meditation may not significantly increase overall synchronization, it enhances network efficiency and integration. As our measurement is on left PFC, it is in line with a previous research on increased prefrontal-limbic connectivity has been linked to improved regulation and reduced stress (Hölzel et al., 2013). In this context, the optimized network structure during meditation may support individual cognitive and emotional functions, contributing to personal resilience and well-being. By enhancing network efficiency and integration, meditation can improve attentional control, regulation, and stress management, qualities especially critical for adolescents in competitive school environments (Goyal et al., 2014).

The distinct impacts of music and meditation on synchronization and network complexity have important implications for social connectedness and emotional resilience in adolescents, particularly in educational settings. Music, by enhancing group synchronization, facilitates shared neural states that promote unity and collective identity. This is facilitated by shared rhythmic stimuli aligning oscillations, fostering unity and collective consciousness (Dai et al., 2022). This heightened synchronization supports stronger social bonds, fostering a sense of belonging and emotional support, which is crucial for resilience in the face of challenges such as academic pressures, social media, and socio-economic stressors and uncertainties (Dikker et al., 2017; Leung and Mu, 2022). These findings underscore the potential of structured auditory stimulation, like high-entropy music, in fostering supportive environments that enhance coping mechanisms and well-being.

Collectively, the findings may underscore the complementary roles of music and meditation in promoting both group-level cohesion and individual cognitive-emotional well-being. This is pertinent in education, where cognitive flexibility and regulation are essential for success and development. From an evolutionary perspective, music’s ability to foster pro-social behavior may have evolved as an adaptive mechanism for group cohesion and cooperation (Kim, 2024). Future research could explore their combined effects, potentially integrating both into curricular interventions aimed at promoting mental health resilience among adolescents.

### 4.1 Limitations and future directions

Limitations include that single-channel EEG limits spatial resolution to explore inter-brain correlation among other brain regions, which is common in dyad hyperscanning studies involving a pair of participants. For a hyperscanning experiment with more than dozens of participants, it is more difficult to balance the number of participants in a network and the number of channels, especially given the 40-min time constraint. Another limitation is the behavioral assessment on group coherence scores, since there has no suitable measurement so far in classroom setting. Longitudinal assessment on hyperscanning EEG and group coherence scores would provide stronger support for the Hebbian theory on social connectivity and learning.

## 5 Conclusion

This study demonstrates the differential effects of 6 Hz high-entropy music and meditation, two commonly used methods in social environment, on group synchronization and network matrices in adolescents. The distinct neural signatures highlight their potential to foster connectedness and resilience through different mechanisms. Music enhances group synchronization and integration, promoting unity and collective states, while meditation, while less prominent, may optimize network efficiency and flexibility, supporting individual cognitive and emotional functions. These neuroscientific findings can contribute to research on music and pro-social behavior. Integrating this evidence into education and therapy can help design efficient intervention for mental health and harmony among adolescents.

## Data Availability

The datasets presented in this article are not readily available because it involves adolescents in the study, please contact the corresponding author for data availability. Requests to access the datasets should be directed to hinhung@hku.hk.

## References

[ref1] Babiloni F. Astolfi L. (2014). Social neuroscience and hyperscanning techniques: past, present and future. Neurosci. Biobehav. Rev. 44, 76–93. doi: 10.1016/j.neubiorev.2012.07.006, PMID: 22917915 PMC3522775

[ref2] Bannan N. Harvey A. R. (2025). Music as a social instrument: a brief historical and conceptual perspective. Front. Cogn. 4:1533913. doi: 10.3389/fcogn.2025.1533913 PMC13281057 42339249

[ref4] Colzato L. S. Barone H. Sellaro R. Hommel B. (2017). More attentional focusing through binaural beats: evidence from the global-local task. Psychol. Res. 81, 271–277. doi: 10.1007/s00426-015-0727-0, PMID: 26612201 PMC5233742

[ref5] Crone E. A. Dahl R. E. (2012). Understanding adolescence as a period of social–affective engagement and goal flexibility. Nat. Rev. Neurosci. 13, 636–650. doi: 10.1038/nrn3313, PMID: 22903221

[ref6] Dai L. Liu Y. Rong F. Yang J. Wang L. Wang B.. (2022). From isolated to connected brains: a systematic review of multi-brain network research with hyperscanning techniques. Brain Sci. 12:15. doi: 10.3390/brainsci12010015, PMID: 35053759 PMC8773655

[ref7] Deco G. Tononi G. Boly M. Kringelbach M. L. (2015). Rethinking segregation and integration: contributions of whole-brain modelling. Nat. Rev. Neurosci. 16, 430–439. doi: 10.1038/nrn3963, PMID: 26081790

[ref8] Dikker S. Wan L. Davidesco I. Kaggen L. Oostrik M. McClintock J.. (2017). Brain-to-brain synchrony tracks real-world dynamic group interactions in the classroom. Curr. Biol. 27, 1375–1380. doi: 10.1016/j.cub.2017.04.002, PMID: 28457867

[ref9] Faber P. L. Milz P. Andrich D. Lehmann D. (2021). The contributions of functional connectivity and spectral power differences to higher intelligence: a thalamocortical model. Intelligence 87:101556. doi: 10.1016/j.intell.2021.101556

[ref10] Fell J. Axmacher N. (2011). The role of phase synchronization in memory processes. Nat. Rev. Neurosci. 12, 105–118. doi: 10.1038/nrn2979, PMID: 21248789

[ref11] Gao X. Cao H. Ming D. Qi H. Wang X. Wang X.. (2014). Analysis of EEG activity in response to binaural beats with different frequencies. Int. J. Psychophysiol. 94, 399–406. doi: 10.1016/j.ijpsycho.2014.10.010, PMID: 25448376

[ref12] Goyal M. Singh S. Sibinga E. M. Gould N. F. Rowland-Seymour A. Sharma R. (2014). Meditation programs for psychological stress and well-being: a systematic review and meta-analysis. JAMA Intern. Med. 174, 357–368. doi: 10.1001/jamainternmed.2013.13018, PMID: others, & Haythornthwaite, J. A 24395196 PMC4142584

[ref13] Grasso-Cladera A. Costa-Cordella S. Mattoli-Sánchez J. Vilina E. Santander V. Hiltner S. E.. (2024). Embodied Hyperscanning for studying social interaction: a scoping review of simultaneous brain and body measurements. Soc. Neurosci., 1–17. doi: 10.1080/17470919.2024.2409758, PMID: 39387663

[ref14] Grootjans Y. Harrewijn A. Fornari L. Janssen T. de Bruijn E. R. A. van Atteveldt N.. (2024). Getting closer to social interactions using electroencephalography in developmental cognitive neuroscience. Dev. Cogn. Neurosci. 67:101391. doi: 10.1016/j.dcn.2024.101391, PMID: 38759529 PMC11127236

[ref15] Hölzel B. K. Hoge E. A. Greve D. N. Gard T. Creswell J. D. Brown K. W. (2013). Neural mechanisms of symptom improvements in generalized anxiety disorder following mindfulness training. NeuroImage 2, 448–458. doi: 10.1016/j.nicl.2013.03.011, PMID: others, & Lazar, S. W 24179799 PMC3777795

[ref16] Huang Z. Zhang Z. Zhou Y. Niu Y. Gupta A. Thakor N.. (2021). Promoting brain functions in online group activities via auditory high-entropy stimulation. Front. Neurosci. 15:754869. doi: 10.3389/fnins.2021.754869

[ref17] Jirakittayakorn N. Wongsawat Y. (2017). Brain responses to a 6-Hz binaural beat: effects on general theta rhythm and frontal midline theta activity. Front. Neurosci. 11:365. doi: 10.3389/fnins.2017.00365, PMID: 28701912 PMC5487409

[ref18] Kim J. (2024). Music and prosocial behavior: a review of the literature. Music Sci. 4. doi: 10.1177/20592043211048935

[ref19] Kropotov J. D. (2009). “Chapter 4- frontal midline Theta rhythm” in Quantitative EEG, event-related potentials and Neurotherapy. ed. Kropotov J. D. (Academic Press), 77–95.

[ref20] Kuyken W. Weare K. Ukoumunne O. C. Vicary R. Motton N. Burnett R. (2013). Effectiveness of the mindfulness in schools Programme: non-randomised controlled feasibility study. Br. J. Psychiatry 203, 126–131. doi: 10.1192/bjp.bp.113.126649, PMID: others, & Huppert, F 23787061

[ref21] Leung C. H. Mu Y. (2022). Spiritual and mental health of teenagers in Hong Kong and in mainland China under the impact of COVID-19. Asian Educ. Dev. Stud. 11, 340–355. doi: 10.1108/AEDS-04-2021-0076

[ref22] Liu J. Q. Zhang R. Q. Geng B. B. Zhang T. Y. Yuan D. Otani S.. (2019). Interplay between prior knowledge and communication mode on teaching effectiveness: interpersonal neural synchronization as a neural marker. NeuroImage 193, 93–102. doi: 10.1016/j.neuroimage.2019.03.004, PMID: 30851445

[ref23] Lomas T. Ivtzan I. Fu C. H. (2015). A systematic review of the neurophysiology of mindfulness on EEG oscillations. Neurosci. Biobehav. Rev. 57, 401–410. doi: 10.1016/j.neubiorev.2015.09.018, PMID: 26441373

[ref24] McWeeny S. Chang L. J. Wheatley T. (2024). Synchronized and anti-phase drumming elicit similar prosociality. Sci. Rep. 12, 1–10. doi: 10.1038/s41598-022-05570-8, PMID: 35110583 PMC8810809

[ref25] Reedijk S. A. Bolders A. Colzato L. S. Hommel B. (2015). Eliminating the attentional blink through binaural beats: a case for tailored cognitive enhancement. Front. Psych. 6:82. doi: 10.3389/fpsyt.2015.00082, PMID: 26089802 PMC4455234

[ref26] Rosenberg A. R. Zhou C. Bradford M. C. Salsman J. M. Sexton K. O'Daffer A.. (2021). Assessment of the promoting resilience in stress management Interventi on for adolescent and young adult survivors of Cancer at 2 years: secondary analysis of a randomized clinical trial. JAMA Netw. Open 4, –e2136039. doi: 10.1001/jamanetworkopen.2021.36039, PMID: 34817581 PMC8613597

[ref27] Sporns O. (2021). The small-world of the cerebral cortex. NeuroImage 238:118209. doi: 10.1016/j.neuroimage.2021.118209 34051354

[ref28] Steinberg L. (2014). Age of opportunity: Lessons from the new science of adolescence. Boston, MA: Houghton Mifflin Harcourt.

[ref29] Waters L. Barsky A. Ridd A. Allen K. (2015). Contemplative education: a systematic, evidence-based review of the effect of meditation interventions in schools. Educ. Psychol. Rev. 27, 103–134. doi: 10.1007/s10648-014-9258-2

[ref30] Wong L. Chan C. (2019). Promotion of adolescent mental health in Hong Kong—the role of a comprehensive child health policy. J. Adolesc. Health 64, S14–S18. doi: 10.1016/j.jadohealth.2019.01.033, PMID: 31122544

[ref31] Wu Y. J. Chien C. L. (2024). The enhancement effect of music-induced positive emotion on altruistic behavior. Front. Psychol. 15:842481. doi: 10.3389/fpsyg.2024.842481

[ref3] Yu L. Du M. (2022). Social networking use, mental health, and quality of life of Hong Kong adolescents during the COVID-19 pandemic. Frontiers in Public Health 10:1040169., PMID: 36388293 10.3389/fpubh.2022.1040169 PMC9659958

[ref32] Zeidan F. Johnson S. K. Diamond B. J. David Z. Goolkasian P. (2010). Mindfulness meditation improves cognition: evidence of brief mental training. Conscious. Cogn. 19, 597–605. doi: 10.1016/j.concog.2010.03.014, PMID: 20363650

