# Supplementary material for: Interbrain synchronization in classroom during high-entropy music listening and meditation: a hyperscanning EEG study
Source: Front Neurosci. 2025 Apr 1;19:1557904. doi: 10.3389/fnins.2025.1557904 (PMC12044879; doi:10.3389/fnins.2025.1557904)
Supplement: Supplementary file 1 [file Data_Sheet_1.docx]

**Supplementary**


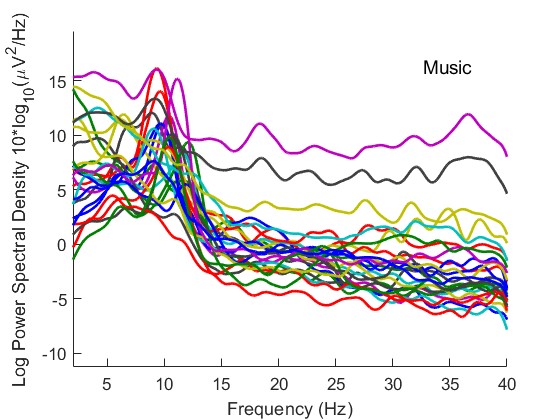


**sFigure 1.** Power spectrum plot of participants in the Music condition.


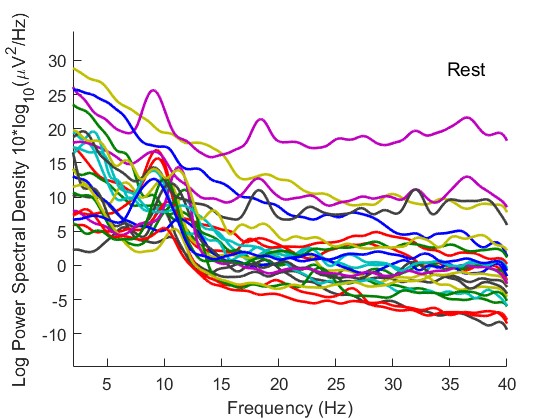


**sFigure 2.** Power spectrum plot of participants in the Rest condition.


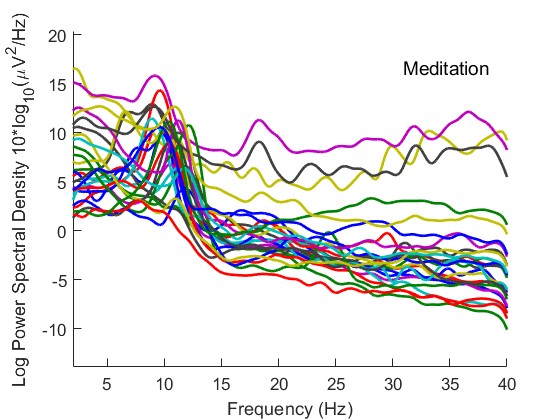


**sFigure 3.** Power spectrum plot of participants in the Meditation condition.


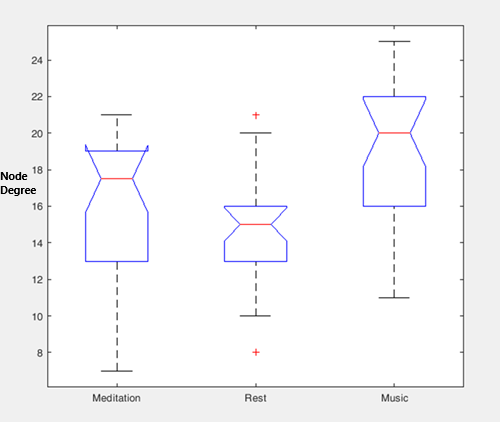


**sFigure 4.** The degrees for the nodes (participants) in a network under different conditions. This graph illustrates how different mental states – meditating, resting, and listening to high-entropy music could lead to distinct patterns of brain connectivity. The quantitative graph-theoretic measures (clustering coefficient and small-world index) confirm that meditation supports a balance of local and global efficiency, while music listening fosters relatively strong overall synchronization, and rest shows the least connectivity among the three conditions.
